# Exploring the Characteristics of Physical Exercise in Students and the Path of Health Education

**DOI:** 10.3389/fpsyg.2021.663922

**Published:** 2021-11-29

**Authors:** Xintong Peng, Lijun Tang

**Affiliations:** ^1^College of Physical Education and Health, Guangxi Normal University, Guilin, China; ^2^Physical Education College, Shanghai Normal University, Shanghai, China

**Keywords:** artificial intelligence, physical exercise, student, health education, smart sports classroom in colleges and universities

## Abstract

College students are taken as the research sample, with the purpose of exploring the characteristics of physical exercise and health education path of students under artificial intelligence (AI) algorithm. First, related literature is studied to understand the physical education system of college students. Then, the current situation of physical exercise of college students is investigated through the interview survey, and the mathematical statistics method is used to analyze the survey results. Moreover, the necessity and paths to carry out health education are discussed through the analysis of the physical exercise behavior of college students. Finally, the college smart sports classroom (SSC) is constructed using AI and the big data analysis method. The experimental results indicate that more than 50% of college students can actively participate in physical exercise. Besides, boys are more likely to take dangerous coping behaviors, while girls are more prone to choose to resist coping behaviors. In addition, there is little difference in age of the distribution of different coping behaviors in physical exercise. Freshmen are more inclined to take risky coping behaviors, and the quantity of students taking resistant coping behaviors increases with the increase of grades. Therefore, relevant physical health education for college students can promote the good habit of health exercise. This study can provide a reliable experimental basis for the development of sports education in the future.

## Introduction

With the rapid development of science and technology today, the material life of people has been greatly improved. Youths are the foundation and driving force of national development, and their healthy growth is vital to the development of the motherland (Stein and Brooks, 2017; Contreras and Vehi, 2018). The knowledge of physical fitness test results, mastery of physical health related knowledge, and learning exercise methods of students do not necessarily improve their physical exercise intentions or behavioral habits. The “conflict of knowledge and action, knowledge but not action” in the physical exercise of students does not exist individually but is a common phenomenon. It reveals that the goal effect of physical education is still very clear from reality, and the declining physical health of students is also extremely prominent (Emanuel and Wachter, 2019). Casey and MacPhail (2018) found that the Ministry of Education and other relevant departments have conducted some surveys on the physical and health organizations of students (6–22 years old) across the country in 1985, 1990, 1995, 2000, 2005, 2010, and 2014 (Casey and MacPhail, 2018). Compared with the survey in 2010, the physical development level (including the height, weight, and bust) of Chinese urban and rural students in 2014 was improved continually. The vital capacity continued to show an upward trend after the rising inflection point in 2010. The detection rate of malnutrition among urban and rural students further decreased, and there was basically no severe or moderate malnutrition. The infection rate of roundworms in rural primary school students continued to decrease. The physical fitness of elementary and middle school students such as speed, flexibility, strength, and endurance continued to show steady improvement. However, there are also some other problems while the overall physical health of students has improved. For example, the overall physical fitness of college students continued to show a downward trend, the detection rate of poor eyesight remained high and continued to show a tendency to younger age, and the obesity detection rate for students of all ages was continuously rising (Verghese et al., 2018). Therefore, reasonable health education for physical exercise of students is extremely important.

At present, the era of information technology (IT) where all things are interconnected and intelligent is coming, and many new information technologies are coming one after another, such as mobile Internet, Internet of Things (IoT), big data, and cloud computing. The Smart Sports Classroom (SSC) is a specific branch of the smart classroom (SC). There is almost no research on the SSC, and no scholars have found a clear conception of the SSC (Bores-García et al., 2021). Generally, the concept of SC can be roughly classified from two perspectives. One is the perspective of smart education, which focuses on educational concepts. With the aid of intelligent information-based teaching equipment, intelligent transformation, and innovation of successful cases of flipped classroom teaching. SC is a new type of digital classroom with the goal of improving student personality, developing student intelligence, improving student quality, and promoting all-around development. The other classification is based on the perspective of smart technology and focuses on educational methods (Davenport and Kalakota, 2019). Through cloud computing, big data, IoT, mobile Internet, and other latest scientific information technologies, an intelligent, interoperable, efficient, and scientific teaching environment can be created. It is a transformation and upgrade of traditional classroom teaching methods, realizing the online push of network classroom resources, and visualized dynamic analysis of learning data, real-time communication between teacher and student, and timely evaluation (Kulikowski, 2019). In the context of the era of wisdom and under the guidance of national policies and the support of many emerging technologies, it is imperative to apply artificial intelligence (AI) and other technologies to the reform of school sports and health education (Nemitz, 2018).

Although SSC has grown mature in theory, there are few cases of its application in the actual classroom. Therefore, taking college students as the research sample, the relevant literature is first studied to understand the physical education system of college students. Second, the questionnaire survey is conducted to investigate the current situation of the physical exercise of college students, and survey results are made mathematical statistics analysis. Furthermore, the necessity and path of health education are discussed based on the analysis of the physical exercise behavior of college students. Ultimately, the university sports fitness center is built by AI and the big data analysis method. The innovation lies in the combination of AI and college physical education curriculum, which improves the quality of the college physical education curriculum and provides new research ideas for the physical and health education of college students.

## Literature Review

### Research Status of Influencing Factors of Physical Health

As an emerging force in the development of a country, it is extremely important for young people to have a healthy body. Physique is the comprehensive and relatively stable characteristics of the quality and structure, physiological functions, and psychological factors of the human body based on heredity and acquisition. Physical fitness usually includes five dimensions, including physical development, physiological function, physical fitness, and athletic ability development, mental development, and adaptability development (Davenport et al., 2020). Physical fitness is a feature of genetic acquisition, but the acquired environment, nutrition, physical exercise, and health care conditions have positive and effective effects on enhancing physical fitness. Czekierda et al. (2017) conducted a systematic review and meta-analysis of the link between the meaning of life and physical health and investigated the types of health indicators, health status (Czekierda et al., 2017), and age of participants as mediating factors. The results showed that there is a weak to moderate correlation between the meaning of life and physical health, with the strongest correlation among the subjective indicators of physical health. In addition, it pointed out the potential role of life meaning in explaining physical health. Hays et al. (2018) assessed the pain intensity in seven health areas [physiological function, fatigue, pain interference (Hays et al., 2018), depressive symptoms, anxiety, ability to participate in social roles and activities, and sleep disorders] by collecting data from the Internet. The results found that the correlation between the PROMIS-29v2.0 physical and mental health score and chronic diseases and other health-related quality of life indicators is in line with the prior assumptions, and preliminary evidence was developed and provided to support the reliability and effectiveness of the PROMIS-29v2.0 physical and mental health score. In addition, it could be used in future research to evaluate the impact of health care interventions and track changes in health over time. White et al. (2019) studied the links between recreational natural contact and self-reported health and well-being over the past 7 days. It was found that compared with those who had no natural contact last week (White et al., 2019), the possibility of reporting good health or high happiness is significantly increased when the contact time was ≥120 min and the positive association peaked between 200 and 300 min per week but did not increase further. Barlett et al. (2020) conducted research related to the physical health of adolescents and completed the measurement of the new adulthood (Barlett et al., 2020), stress, gender, and physical health symptoms of adolescents. It was found that the perception of emerging adult dimensions representing unsuccessful transitions (negative/unstable) has a positive predictive effect on stress and physical health concerns, but positive adult transition dimensions (experiments/possibility) have negative predictions for these results.

### Current Status of Research on the Correlation Between Physical Exercise and Physical Health of Teenagers

The correlation between physical fitness and physical exercise has received more research attention. Bebeley et al. (2017) scored the physical exercise motivation level of college students by measuring and evaluating the factors under physical exercise self-efficacy (PESE) of college students (Bebeley et al., 2017). It turned out that under the PESE, the vast majority of subjects reacted more to “I can achieve my exercise intention even when I’m busy,” which meant the interviewees are willing to engage in physical exercise even in the busy schedule. LaCount and Hartung (2018) initially supported the effect of physical exercise on attention deficit hyperactivity disorder (ADHD) and common injury sites through physical exercise studies on samples of children and adolescents. In reviewing studies of more substantial physical exercise in adults with depression (LaCount and Hartung, 2018), it was found that light to moderate-intensity aerobic exercise and high-intensity intermittent aerobic exercise are most likely to better the emotion to a great degree, thereby increasing the long-term participation of emerging adults. Although there are not many studies on the effectiveness of physical exercise for ADHD in just adults, there are still many reasons for mental health providers to recommend it as an adjunct to psychosocial and drug treatment. Peeters et al. (2019) studied the perception of vibration signals in physical exercise by comparing the recognition difference between fixed posture and bicycle posture. The results showed that the fixed posture of the thigh and spine is similar to the correct recognition of physical exercise (Peeters et al., 2019), and the vibration signal of the spine is more accurate and faster, which means that vibration feedback also has the potential application prospects in physical exercises, such as cycling. Valenti et al. (2020) found that during physical exercise, the release of different growth factors, cytokines, and hormones have positive impacts on the functions of the heart, bone, brain, and skeletal muscle, and that physical activity may stimulate tissue remodeling (Valenti et al., 2020).

In summary, there are many studies on the physical exercise of students, and most of them analyze the impacts of physical exercise on physical health. However, the research on the intervention of physical exercise from the perspective of health education is relatively rare. Therefore, the current status of physical exercise of students is analyzed, and AI technology is applied in the health education and exercise of students, which exerts an extremely important role for the healthy development of adolescents.

### Necessity of Applying Artificial Intelligence in Sports Classroom

Investigating the current status of physical exercise of college students is conducive to understanding the importance of the lifelong sports concept of college students and the formation of good daily physical exercise habits. The sports classroom can play a guiding role in the physical exercise of students. The necessity of applying AI to sports classrooms can be analyzed from multiple perspectives such as students, society, and schools (Golden, 2017; Huang and Rust, 2018; Nichols et al., 2019; Calvano et al., 2020).

From the perspective of social development, the construction of SSC in colleges and universities is to use the Internet, big data, AI, and other technologies to intelligently transform and innovate the college sports classrooms. The innovation of technology and teaching methods can provide powerful support for digital classrooms and smart campuses, contributing to the development of the smart society of China (André et al., 2018; Krittanawong et al., 2018). SSC in colleges and universities is imperative from the perspective of the construction needs of a smart society, and it is reforming and innovating teaching evaluation through smart means from the perspective of physical education. As a systematic project, it is helpful to fully stimulate the vitality of the development of education, effectively promote the collaborative innovation of industry, and actively implement the innovation-driven development strategy. It is the only way to reform the physical education curriculum. From the perspective of students, physical exercise is the most direct measure to enhance physical fitness, and physical health is the foundation of student development. SSC can stimulate the interest of college students in physical exercise, form correct physical exercise habits and lifelong physical education awareness, and improve physical fitness (Stead, 2018). Then, it can further hone strong will, cultivate good morality and fighting spirit, and establish a correct outlook on life, values, and world outlook. Therefore, the construction of SSC in colleges and universities should not be delayed from the perspective of the needs of students in physical and mental development (Maddox et al., 2019).

### Review of Related Works

At present, AI technology is widely used in the field of educational technology, including learning platforms, learning environments, and learning tools. Big data, a terminology of the IT industry, refers to the data set that cannot be captured, managed, and processed by conventional software tools in a certain time range. It is a massive, high-growth, and diversified information asset that requires new processing modes to acquire stronger capabilities of decision-making, insight, and process optimization. The hot research areas are introduced as follows, including intelligent learning platforms, intelligent learning environment, and intelligent auxiliary teaching tools. (1) Intelligent learning platform: online learning platforms represented by massive open online courses (MOOC) can record massive data of the learning process of learners. Based on data analysis, the platform can accurately grasp the learning status of each learner and then provide personalized teaching diagnoses and teaching recommendations for learners. Accordingly, numerous studies based on learner behavior data have gradually become research hotspots, i.e., predicting academic performance based on learning behavior characteristics of learners. (2) Intelligent learning environment: intelligent learning environment includes smart campus, smart classroom, and smart library. Smart campus integrates teaching resources and applications through network information technology and AI technology and realizes the smart and scientific decision-making of campus management. For example, the smart campus system designed by Professor Uskov of Bradley University covers the teaching environment, teaching management, and teaching process. Smart classrooms use advanced information technology, network technology, computer technology, and AI technology to provide multiple data support for various decisions of classroom teaching activities, such as learner type identification and utility analysis of content display methods. (3) Intelligent auxiliary teaching tool: intelligent auxiliary teaching tool is an intelligent system that simulates the role of human teachers and assists learners to learn, such as an intelligent teaching system and teaching robots. Intelligent auxiliary teaching tools can continuously evaluate the learning state or learning ability of learners in the process of learning knowledge, and implement precise intervention according to their state.

In general, the digital detection of SSC mainly combines intelligent bracelets and other equipment with background data software to establish the sports health records of each student. Parents can know the movement of the children through the data at any time. Besides, schools can find the shortcomings of physical education timely to make improvements and detect whether the students have discomfort during sports exercise. In addition, systematic data helps schools understand the situation of students and the quality of sports at any time. The complex date operation of the background tends to be more accurate with the continuous advancement of SSC. Different background system operation modes have their own characteristics, but in general, they have a relatively unified idea.

Browser/Severe (B/S) architecture is the architecture mode combining browsers and servers, which is a transformation and improvement of Client/Severe architecture with the rise of Internet technology. In the B/S framework, the user work interface is realized through the World Wide Web (WEB) browser. A few transaction logics are implemented in the front end (Browser) while the main transaction logic is implemented in the Server, forming a three-tier structure. B/S architecture is a network architecture model after the rise of WEB, and the WEB browser is the most important application software of the client. This pattern unifies the client, concentrates the core part of the system function realization to the server, and simplifies the development, maintenance, and usage of the system. The B/S architecture simply requires a browser on the client, such as Netscape Navigator or Internet Explorer, and a database in the Server, such as Oracle, Sybase, Informix, or SQL Server. The data interaction between the browser and the database is realized through Web Server, which greatly simplifies the client computer load, reduces the cost and workload of system maintenance and upgrading, and reduces the overall cost of users. Virtual Reality (VR) is a new practical technology developed in the twentieth century, containing a computer, electronic information, and simulation technology. It is basically achieved by the simulation of the computer of the virtual environment, which gives people a sense of environmental immersion. With the continuous development of social productivity and science and technology, the demand for VR technology is growing in all walks of life. VR technology has also made great progress and has gradually become a new field of science and technology.

## Analytical Method

### Investigation and Research Design for Physical Exercise of Students

#### Research Methods

The literature search method (Agrawal et al., 2019) is adopted. Many domestic and foreign monographs, master (doctoral) dissertations, literature results, journals, and magazines related to haze air pollution and physical exercise response behaviors of college students are referred in the early stage, so as to analyze the theoretical background and significance of the research question and summarize the current research status. In addition, the interview survey is conducted on experts and scholars, college physical education teachers, and college students, according to the research needs and technical needs (Kanagasingam et al., 2018), to learn the cognitive level of college students on haze weather, their attitudes of physical exercise, and their physical coping behaviors of exercise. The interview is implemented through a series of questionnaires on individuals to understand their views on air pollution, and their physical exercise status, and sports psychology, to find out the relationship between them (Adamson and Smith, 2018). Then, the result of the questionnaire survey is analyzed comprehensively. Based on the material and analysis results of the interview content and previous research and theories, expert reliability and validity questionnaire on air pollution-related issues and physical exercise response behaviors are compiled which is answered by relevant experts. For experts and students to determine the accuracy and comprehensibility of the structure and description of the questionnaire, at least 20 experts and students can be selected, respectively, to put forward the personal opinions and suggestions on the structure and content design of the questionnaire for further modification. The questionnaire is processed for structural validity and internal consistency reliability. The data test results of the haze pollution perception level and physical exercise response behavior are compared, and the factor analysis and correlation analysis are completed, aiming to scientifically construct the theoretical basis for the haze perception level and physical exercise response behavior of college students.

#### Questionnaire Design

The sample data is collected through the questionnaire survey to evaluate the physical exercise of students. During the questionnaire design (Appendix Table A1), the haze perception scale of college students is first combined with the physical exercise coping behavior scale. Then, the questionnaire is divided into three parts: basic information, the scale, and the open problem. It investigates the perception of college students on haze weather, the influence of haze on the outdoor physical exercise of college students, and the behavior choice of physical exercise of college students under haze weather. Coping style refers to the cognitive and behavioral patterns adopted by individuals in the face of setbacks and pressures, which can also be called coping strategies or coping mechanisms. It is an important mediating factor in the process of psychological stress. Individual coping style affects the nature and intensity of stress response and then regulates the relationship between stress and stress results. Previous researchers mainly used two different conceptual models in the process of exploring stress response, namely, the ego psychology model and the relation psychology model. Among them, the ego psychology model mainly revolves around the theme of psychological development and regards coping style as an aspect of personality based on the concept of psychological defense. The relation psychology model regards coping as a dynamic process of a series of constantly changing thinking and actions used by individuals to deal with internal or external requirements, during which individuals often adopt a combination of different coping styles or strategies.

In the questionnaire, the value of exercise intensity is represented by the common physiological indicators, such as heart rate. Generally, it is believed that 120 times/min represents small exercise intensity, 120–150 times/min denotes medium exercise intensity, and 150–180 times/min or more than 180 times/min signifies large exercise intensity. The options of some questions on the scale are based on the Likert Score Sum Scale, among which 1–5 reveals “totally disagree,” “basically disagree,” “neither disagree nor agree,” “basically agree,” and “totally agree,” respectively. This questionnaire does not involve personal privacy in the whole process of questionnaire design, distribution, and data collection. Besides, the whole questionnaire survey is conducted with the consent of participants (not less than 18 years old). This survey has been approved by the leadership of higher vocational colleges and relevant departments. The questionnaire is not open to the public and is only used for research purposes (Hoey et al., 2018).

#### Data Statistics and Analysis

An equal amount of sampling questionnaires is issued in three representative undergraduate universities to reduce the deviation of the survey results and improve the strictness, scientificity, and representativeness of the questionnaire. A total of 321 questionnaires are distributed and 309 are recovered, with a recovery rate of 96.26%. Therefore, the questionnaire survey is effective. After reviewing the returned questionnaire and eliminating the questionnaire with obvious defects to ensure the validity of the questionnaire, there are, ultimately, 285 valid questionnaires in total. Cronbach’s α coefficient is a statistic of the average value of the half reliability coefficient obtained by all possible project division methods of the scale, which is the most common reliability measurement method. It was first named by American educator Lee Cronbach in 1951. If a scale has n questions and the average correlation coefficient between questions is r, the standardized α coefficient of this scale is given as follows:


(1) α=n⁢r/[(n-1)⁢r+1]

The questionnaire is evaluated by Cronbach’s α value to verify the reliability, stability, and index system of the physical exercise questionnaire of college students and to test the reliability coefficient of the questionnaire (Noorbakhsh-Sabet et al., 2019). The internal consistency of the score of each question in the questionnaire is verified. Additionally, IBM SPSS 24.0 is used to evaluate the α reliability coefficient. The results indicate that the average Cronbach α of the questionnaire is 0.830. Therefore, the designed questionnaire has strong internal consistency, stability, and high reliability, which is reasonable and effective. This questionnaire has reliable research significance.

### Analysis on the Construction Path of Applying Artificial Intelligence in the Sport Classroom

The characteristics of physical exercise of college students are investigated and analyzed. In the physical education class, the “health first” is adhered to while implementing the teaching concept of “jogging.” Besides, mobile internet, Internet of Things, big data, and cloud computing are integrated to innovate the physical education model further, enhance the initiative of students in learning, improve teaching efficiency, visualize physical education data, organically integrate both inside and outside classes, and promote exchanges among students, teachers, parents, and schools (Wang et al., 2019).

In the SSC built by universities, the overall structure of the intelligent service platform takes humanity as the core through the intelligent terminal equipment platform, the stadium intelligent platform, and the health cloud management platform. AI equipment supports powerful background data analysis, processing, storage, and extraction, and organically integrates students, teachers, parents, experts, scholars, and multiple elements, so that smart equipment terminals and stadium equipment are combined. The information platform connected to the health cloud management platform, stadium intelligent platform, and intelligent terminal equipment platform provides SSC with an intelligent, informatized, and integrated service support environment. In the university logistics center, the function of the health cloud management platform is like the brain of a person, which is the cloud intelligent database of the university logistics center. Cloud computing and other science and technology are adopted to collect, store, and control SSC data in colleges and universities. The data are collected through terminal equipment and transmitted to the cloud platform for processing and analysis. Finally, relevant information about student physical exercise is given.

### Logic Framework of the Smart Analysis System

**FIGURE 1:**
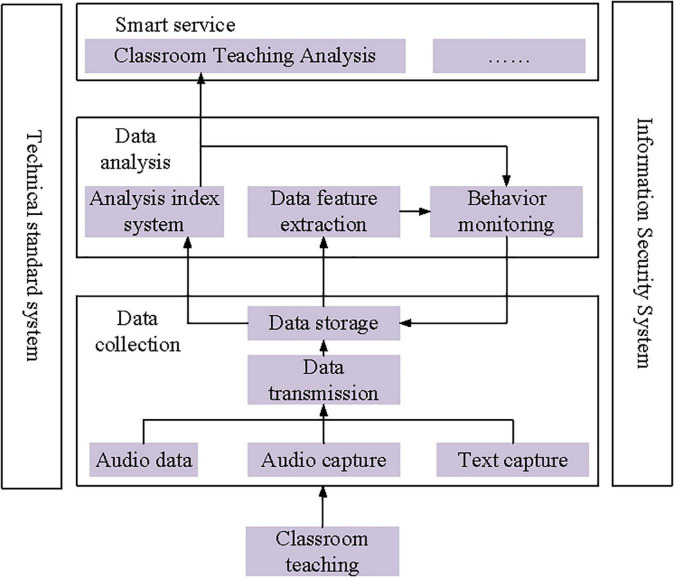
Logic framework of the system.

The process of classroom teaching analysis includes the collection, representation, analysis, and display of teaching behavior data, which needs to be realized through the application system. A logical framework of a smart classroom teaching behavior analysis system is designed to rationalize the feasibility of the research method while positioning and combing the research elements and the relationship between elements. The framework takes classroom teaching behavior analysis as the goal, covering the acquisition, storage, modeling, processing, and analysis involved in the analysis process, along with the supportive environment as an application system. The logic architecture of the system is divided into five parts: data acquisition module, data analysis module, smart service module, the system supporting technical standards, and information security module. Figure 1 indicates the logic framework of the system, involving the main research object, method, and modules.

## Results and Discussion

### Investigation Results and Analysis for Physical Exercise of Students

**FIGURE 2:**
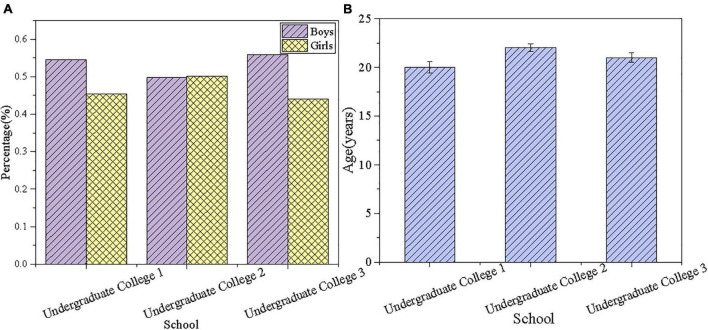
Descriptive analyses of respondents (**A**: gender ratio; **B**: age distribution).

Figure 2 reveals the general descriptive analysis of respondents.

Figure 2A indicates that the gender ratio of the respondents in three undergraduate universities is relatively balanced. In detail, there are more male students than female students in the first and third undergraduate universities. Correspondingly, male students are slightly more than female students among the respondents. Figure 2B shows the age distribution of the respondents, where the age of the respondents in the second undergraduate university is slightly higher than that in the first and third undergraduate universities. However, the respondents in the three undergraduate universities are all about 20 years old, who are between 20 and 24 years old, which is consistent with the general age distribution of college students.

**FIGURE 3:**
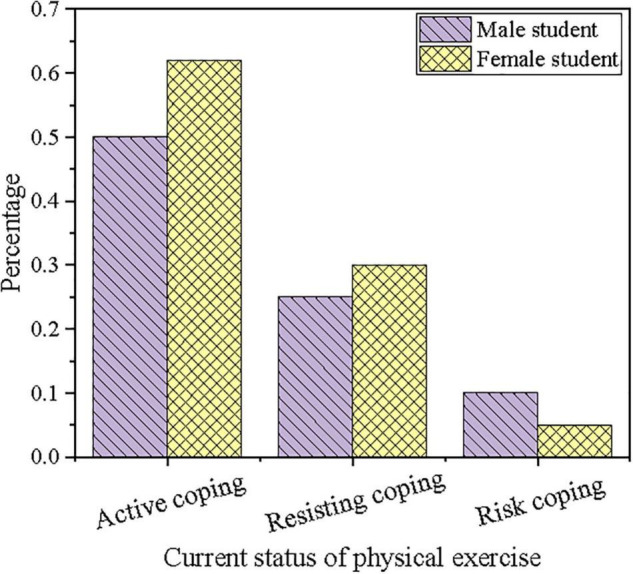
Comparison on coping attitude to physical exercise of students in different genders.

**FIGURE 4:**
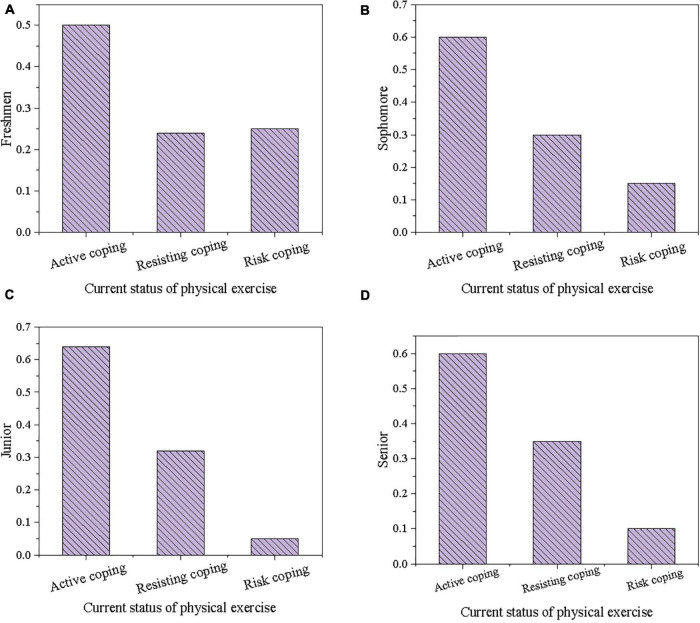
Comparison results of different grades of coping attitudes of college students toward physical exercise. (**A**: Freshmen; **B**: Sophomore; **C**: Junior; **D**: Senior).

The current status of physical exercise of students is investigated by questionnaire survey, and the results are shown in Figures 3, 4.

Figure 3 denotes the comparison of different coping attitudes to physical exercise of students of different genders. The results show that no matter male or female students, more than half of the students are willing to actively carry out physical exercise under the haze pollution with appropriate protective measures and coping behavior. Besides, female students may choose more positive coping behavior, while more male students tend to choose risk coping behavior. This indicates that male students are more likely to ignore the air environment to enjoy sports when doing outdoor exercise. Therefore, a comprehensive analysis shows that although more than half of the students are willing to take a positive attitude in the haze weather, the countermeasures and behavior of physical exercise are not ideal in the haze weather. Furthermore, they may be extremely negative or may take risk coping behavior.

Figure 4 displays the comparison results of different grades of coping attitudes of college students toward physical exercise. There is not much difference in the coping behavior of college students of different grades in physical exercise. In terms of resisting coping behaviors, there is basically a gradual increase from freshman to senior year, which may be due to the increase in grade, already greater academic or work pressure, and the reduced energy expenditure on physical exercise, and the chance of resisting and responding increases in face of haze. Analysis on the choice of risk coping behaviors discloses that freshman students are more likely to exercise in a haze environment without taking any protective or countermeasures than other grade students. It further indicates that freshman students need more knowledge about haze protection and good physical exercise to guide the intervention of behavioral habits.

Furthermore, the correlation between the normal exercise intensity of college students and different physical exercise coping behaviors is analyzed, and the results are shown in Table 1. Data in the figure come from the questionnaire. Students who choose active physical exercise coping behaviors usually follow the range of exercise intensity from low to sustained intensity. Students with sustained moderate-intensity account for the highest proportion, reaching about 50%. Students who choose to resist coping behavior usually do not take physical exercise, participate in light physical exercise, or participate in unsustainable high-intensity physical exercises, such as playing football, basketball, and tennis. However, students who choose risk response behaviors usually engage in unsustainable high-intensity physical exercises. Therefore, facing haze threat. Students who do not usually participate in physical exercise still choose to resist the coping behavior. However, students who usually have small or medium-intensity exercise habits mainly choose positive coping behaviors. In contrast, students who usually have sustainable exercise habits are more likely to choose risk coping behaviors.

**TABLE 1:** Correlation between the physical exercise coping behaviors and exercise intensity of college students.

Exercise intensity	Active coping	Resisting coping	Risk coping
No participation	1.53	23.62	6.52
Slight	10.08	29.53	16.27
Low intensity	20.36	11.47	12.08
Sustained medium strength	29.61	5.86	9.45
High intensity of not lasting	20.87	23.75	49.86
Long lasting strength	17.61	5.77	5.82

By investigating the status quo of physical exercise of college students and the corresponding statistical analysis, the perception level, gender difference, age difference, and physical exercise habits of college students are all related to the coping behavior of the physical exercise of college students. Results suggest that girls do better than boys in haze protection, but some girls resist physical exercise to prevent haze damage. Boys are less concerned about choosing corresponding protective gears or adjusting the original planned physical exercise content based on air quality than girls, forming a typical physical exercise risk response behavior. The characteristics of boys and girls in physical exercise coping behavior choices can be judged from gender differences. The number of students who choose to resist coping behavior increases with grades. Usually, juniors and seniors no longer need to choose compulsory physical education, making daily physical exercise motivation decline. When the weather or environment is slightly unfavorable, it is easy to resist. Therefore, the physical exercise coping behavior of college students in the face of haze is also related to their usual exercise habits. It is essential to cultivate the concept of lifelong physical education for college students and to develop good daily physical exercise habits.

### Artificial Intelligence Physical Exercise Effect Analysis

**FIGURE 5:**
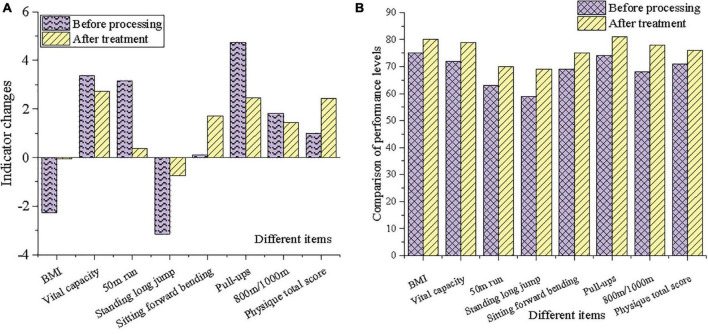
Artificial Intelligence (AI) physical exercise effect analysis (**A**: index change; **B**: performance level).

As shown in Figure 5, several conventional physical exercise tests are analyzed. Among them, 800 m/1,000 m, total physical fitness score has a predictive effect on theoretical performance. Sitting forward bends, 800 m/1,000 m, sit-ups/pull-ups, and total physical fitness scores have predictive effects on technical performance. Sitting forward bends, 800 m/1,000 m, sit-ups/pull-ups, and total physical fitness scores have predictive effects on overall performance. Through AI courses, physical exercise behavior can effectively improve student performance, proving the effectiveness of the proposed approach.

Figure 5 shows the results of the comparison of physical exercise effects of different system models. Compared with previous studies, the proposed approach involves the latest sports health training systems, including B/S architecture, deep learning, and virtual reality. In the sports system, the results before and after the sports performance score are compared. As a result, the proposed system has obvious advantages in method, and student performance has improved significantly.

**FIGURE 6:**
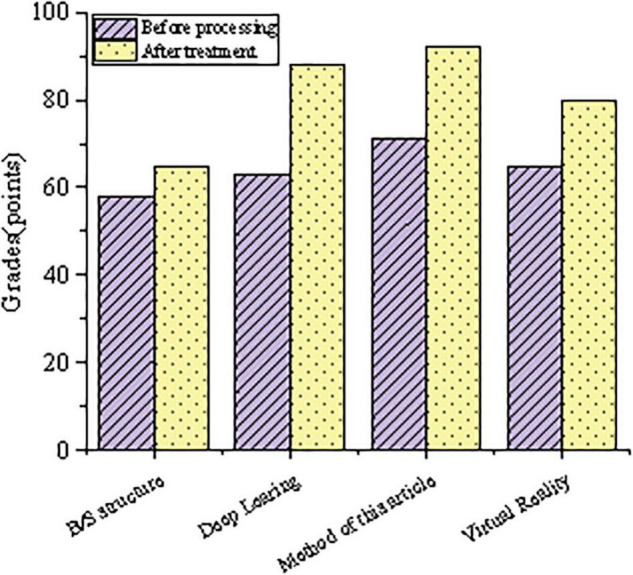
Comparison results of physical exercise effects of different system models.

The comparison results of physical exercise effects of different system models are shown in Figure 6.

The path of physical education in colleges and universities is as follows. First, define the level of sports resources in colleges and universities and formulate different levels of development planning. According to the proportion of sports resources, colleges and universities can be divided into upper sports resources schools, middle sports resources schools, and lower sports resources schools. Schools with different levels should adopt different development strategies. The upper sports resource schools mainly adopt the “independent” development strategy, and the education authorities only formulate various development standards and development indicators for schools to fully mobilize the enthusiasm and initiative of the school, which formulate its development planning and implementation measures independently. Middle sports resource schools adopt the development strategy of “joint promotion.” The main function of the education department is to plan and regulate its strategy, provide support, and help for its insufficient sports resources, and encourage schools to actively cooperate with enterprises and communities in manpower, material, and financial sports resources.

Second, exploring distinctive sports resources in colleges and universities in the region, and actively developing specific sports courses. With a vast territory, there is a huge gap in the development of various regions in China due to various reasons such as history, resources, and geographical location. Therefore, China is often divided into several different regions to facilitate the management and distinction according to different division methods. For example, by administrative region, China is divided into East China, South China, Central China, North China, Northwest China, and Southwest China. By economic zone, the country is divided into the eastern coastal area, central inland area, and western remote area. According to the six comprehensive economic zones, the whole country includes the Northeast China Economic Zone, Economic Zone of Middle Yellow River, Economic Zone in the Middle and Lower Reaches of the Yangtze River, Southeast Coastal Economic Zone, Southwest Comprehensive Economic Zone, and Northwest Economic Zone.

Last, accurately grasping the sports needs and hobbies of different classes of college students and enhancing the particularity of physical education. Under the background of the growing differentiation of social classes, is there any class difference in fitness needs and hobbies among people of different social classes? According to the investigation and study of relevant information, there are certain differences in the sports life of members of all social strata. These differences are reflected in the sports population, sports activities, sports venues, and sports consumption level. Statistics based on the China Health and Nutrition Survey show that like social stratification, sports participation groups are also stratified, and people at different levels have different sports participation preferences. There are demographic differences in the sports participation of residents, such as gender, place of residence, education level, occupation type, and income level.

## Conclusion

In summary, a questionnaire survey on the current status of physical exercise of college students reveals that more than half of college students can actively participate in physical exercise under the premise of ensuring that their health is not threatened in the face of haze, but the characteristics of their physical exercise behaviors are not certainly beneficial to health. Thus, AI technology is adopted to build the SSC in colleges and universities, which can make the physical exercise behaviors of students more active and develop healthy physical exercise habits, providing experimental references for the later development of physical education. However, there are also some shortcomings that are worth noting in future research. The technologies such as the Internet, the IoT, and big data on which the development of SSC relies are also developing and improving. Therefore, the construction of SSC is a dynamic process. In addition, it is necessary to constantly update the technology, and put forward the corresponding teaching mode and construction path in different classroom construction stages. Moreover, the teaching mode can further practice SSC. The experimental intervention of the SSC control group and an experimental group can be set to analyze the teaching effect of SSC attitude and behavior, and continuously improve the physical education mode.

## Data Availability Statement

The raw data supporting the conclusions of this article will be made available by the authors, without undue reservation.

## Ethics Statement

The studies involving human participants were reviewed and approved by the Shanghai Normal University Ethics Committee. The patients/participants provided their written informed consent to participate in this study. Written informed consent was obtained from the individual(s) for the publication of any potentially identifiable images or data included in this article.

## Author Contributions

Both authors listed have made a substantial, direct, and intellectual contribution to the work, and approved it for publication.

## Conflict of Interest

The authors declare that the research was conducted in the absence of any commercial or financial relationships that could be construed as a potential conflict of interest.

## Publisher’s Note

All claims expressed in this article are solely those of the authors and do not necessarily represent those of their affiliated organizations, or those of the publisher, the editors and the reviewers. Any product that may be evaluated in this article, or claim that may be made by its manufacturer, is not guaranteed or endorsed by the publisher.

## Appendix

**TABLE A1:** Questionnaire on influencing factors of physical exercise.

	Totally disagree	Basically disagree	Basically agree	Relatively agree	Totally agree
1. The school grounds and equipment are not perfect, so I often go outside to exercise.					
2. There are fewer people exercising at school and I don’t want to exercise either.					
3. I have no time to exercise because of the academic pressure.					
4. I like PE class.					
5. I like the way the PE teacher teaches.					
6. My parents prefer sports.					
7. My parents may urge me to take part in physical exercise.					
8. My parents may take part in sports exercise with me.					
9. My parents only remind me to study; they don’t support me to go out to exercise.					
10. Personally, I prefer sports.					
11. I think physical exercise can make me happier and sunny.					
12. I think physical exercise can improve the efficiency of study.					
13. I spend more time exercising than playing mobile games.					
14. I have developed the habit of exercising to a certain extent.					
15. I think our government attaches more importance to sports.					
16. I think there are a lot of people exercising in society.					
17. I live in a neighborhood where I can see propaganda about sports knowledge.					
18. Where I live, there are sports fields or fitness facilities.					
19. As I know, there have been some sporting events around where I live.					
20. I buy the necessary sports equipment every year.					

*Please remark “√” below the corresponding options.*

## References

[B1] Adamson A. S. Smith A. (2018). Machine learning and health care disparities in dermatology. *JAMA Dermatol.* 154 1247–1248. 10.1001/jamadermatol.2018.2348 30073260

[B2] Agrawal A. Gans J. S. Goldfarb A. (2019). Artificial intelligence: the ambiguous labor market impact of automating prediction. *J. Econ. Perspect.* 33 31–50. 10.1257/jep.33.2.31

[B3] André Q. Carmon Z. Wertenbroch K. Crum A. Frank D. Goldstein W. (2018). Consumer choice and autonomy in the age of artificial intelligence and big data. *Custom. Needs Solut.* 5 28–37. 10.1007/s40547-017-0085-8

[B4] Barlett C. P. Barlett N. D. Chalk H. M. C. (2020). Transitioning through emerging adulthood and physical health implications. *Emerg. Adulthood* 8 297–305.

[B5] Bebeley S. J. Liu Y. Wu Y. (2017). Physical Exercise Self-Efficacy for College Students’ Level of Motivation In Physical Activity. *Int. J. Sci. Res.* 6 81–85.

[B6] Bores-García D. Hortigüela-Alcalá D. Fernandez-Rio F. J. González-Calvo G. Barba-Martín R. (2021). Research on cooperative learning in physical education: systematic review of the last five years. *Res. Q. Exerc. Sport* 92 146–155. 10.1080/02701367.2020.1719276 32023176

[B7] Calvano E. Calzolari G. Denicolo V. Pastorello S. (2020). Artificial Intelligence, Algorithmic Pricing, and Collusion. *Am. Econ. Rev.* 110 3267–3297.

[B8] Casey A. MacPhail A. (2018). Adopting a models-based approach to teaching physical education. *Phys,. Educ. Sport Pedagogy* 23 294–310. 10.1080/17408989.2018.1429588

[B9] Contreras I. Vehi J. (2018). Artificial intelligence for diabetes management and decision support: literature review. *J. Med. Int. Res.* 20:e10775. 10.2196/10775 PMC6000484 29848472

[B10] Czekierda K. Banik A. Park C. L. Luszczynska A. (2017). Meaning in life and physical health: systematic review and meta-analysis. *Health Psychol. Rev.* 11 387–418. 28488471 10.1080/17437199.2017.1327325

[B11] Davenport T. Guha A. Grewal D. Bressgott T. (2020). How artificial intelligence will change the future of marketing. *J. Acad. Market. Sci.* 48 24–42. 10.1007/s11747-019-00696-0

[B12] Davenport T. Kalakota R. (2019). The potential for artificial intelligence in healthcare. *Fut. Healthcare J.* 6:94. 10.7861/futurehosp.6-2-94 31363513 PMC6616181

[B13] Emanuel E. J. Wachter R. M. (2019). Artificial intelligence in health care: will the value match the hype? *JAMA* 321 2281–2282. 10.1001/jama.2019.4914 31107500

[B14] Golden J. A. (2017). Deep learning algorithms for detection of lymph node metastases from breast cancer: helping artificial intelligence be seen. *JAMA* 318 2184–2186. 10.1001/jama.2017.14580 29234791

[B15] Hays R. D. Spritzer K. L. Schalet B. D. Cella D. (2018). PROMIS ^®^ -29 v2.0 profile physical and mental health summary scores. *Qual. Life Res.* 27 1885–1891. 10.1007/s11136-018-1842-3 29569016 PMC5999556

[B16] Hoey J. Schröder T. Morgan J. Rogers K. B. Rishi D. Nagappan M. (2018). Artificial intelligence and social simulation: studying group dynamics on a massive scale. *Small Group Res.* 49 647–683. 10.1177/1046496418802362

[B17] Huang M. H. Rust R. T. (2018). Artificial intelligence in service. *J. Serv. Res.* 21 155–172.

[B18] Kanagasingam Y. Xiao D. Vignarajan J. Preetham A. Tay-Kearney M.-L. (2018). Evaluation of artificial intelligence–based grading of diabetic retinopathy in primary care. *JAMA Netw. Open* 1:e182665. 10.1001/jamanetworkopen.2018.2665 30646178 PMC6324474

[B19] Krittanawong C. Bomback A. S. Baber U. Bangalore S. Messerli F. H. Wilson T. W. H. (2018). Future direction for using artificial intelligence to predict and manage hypertension. *Curr. Hypertension Rep.* 20:75. 10.1007/s11906-018-0875-x 29980865

[B20] Kulikowski C. A. (2019). Beginnings of artificial intelligence in medicine (AIM): computational artifice assisting scientific inquiry and clinical art–with reflections on present aim challenges. *Yearbook Med. Inform.* 28:249. 10.1055/s-0039-1677895 31022744 PMC6697545

[B21] LaCount P. A. Hartung C. M. (2018). Physical exercise interventions for emerging adults with attention-deficit/hyperactivity disorder (ADHD). *ADHD Rep.* 26 1–11. 10.1521/adhd.2018.26.5.1

[B22] Maddox T. M. Rumsfeld J. S. Payne P. R. O. (2019). Questions for artificial intelligence in health care. *JAMA* 321 31–32. 10.1001/jama.2018.18932 30535130

[B23] Nemitz P. (2018). Constitutional democracy and technology in the age of artificial intelligence. *Philos. Transac. R. Soc. A Mathemat. Phys. Eng. Sci.* 376:20180089. 10.1098/rsta.2018.0089 30323003

[B24] Nichols J. A. Chan H. W. H. Baker M. A. B. (2019). Machine learning: applications of artificial intelligence to imaging and diagnosis. *Biophys. Rev.* 11 111–118. 30182201 10.1007/s12551-018-0449-9 PMC6381354

[B25] Noorbakhsh-Sabet N. Zand R. Zhang Y. Abedi V. (2019). Artificial intelligence transforms the future of health care. *Am. J. Med.* 132 795–801. 10.1016/j.amjmed.2019.01.017 30710543 PMC6669105

[B26] Peeters T. van Breda E. Saeys W. Schaerlaken E. Vleugels J. Truijen S. (2019). Vibrotactile feedback during physical exercise: perception of vibrotactile cues in cycling. *Int. J. Sports Med.* 40 390–396. 10.1055/a-0854-2963 30965375

[B27] Stead W. W. (2018). Clinical implications and challenges of artificial intelligence and deep learning. *JAMA* 320 1107–1108. 10.1001/jama.2018.11029 30178025

[B28] Stein N. Brooks K. (2017). A fully automated conversational artificial intelligence for weight loss: longitudinal observational study among overweight and obese adults. *JMIR Diabetes* 2:e28. 10.2196/diabetes.8590 30291087 PMC6238835

[B29] Valenti M. T. Dalle Carbonare L. Dorelli G. Mottes M. (2020). Effects of physical exercise on the prevention of stem cells senescence. *Stem Cell Rev. Rep.* 16 33–40. 10.1007/s12015-019-09928-w 31832933

[B30] Verghese A. Shah N. H. Harrington R. A. (2018). What this computer needs is a physician: humanism and artificial intelligence. *JAMA* 319 19–20. 10.1001/jama.2017.19198 29261830

[B31] Wang F. Casalino L. P. Khullar D. (2019). Deep learning in medicine—promise, progress, and challenges. *JAMA Internal Med.* 179 293–294. 10.1001/jamainternmed.2018.7117 30556825

[B32] White M. P. Alcock I. Grellier J. Wheeler B. W. Hartig T. Warber S. L. (2019). Spending at least 120 minutes a week in nature is associated with good health and wellbeing. *Sci. Rep.* 9 1–11. 10.1038/s41598-019-44097-3 31197192 PMC6565732

